# Brain Glutamate Dynamics Predict Positive Agency in Healthy Women

**DOI:** 10.21203/rs.3.rs-3021527/v1

**Published:** 2023-06-15

**Authors:** Tara L. White, Meghan A. Gonsalves, Ashley D. Harris, Edward G. Walsh, Hannah E. Joyce

**Affiliations:** Brown University; Brown University; University of Calgary

## Abstract

Contributions of brain glutamate to conscious emotion are not well understood. Here we evaluate the relationship of experimentally-induced change in neocortical glutamate (ΔGlu) and subjective states in well individuals. Drug challenge with *d*-amphetamine (AMP; 20 mg oral), methamphetamine (MA; Desoxyn^®^, 20 mg oral), and placebo (PBO) was conducted on three separate test days in a within-subjects double blind design. Proton magnetic resonance spectroscopy (MRS) quantified neurometabolites in the right dorsal anterior cingulate cortex (dACC) 140–150 m post-drug and PBO. Subjective states were assessed at half hour intervals over 5.5-hours on each session, yielding 3,792 responses per participant (91,008 responses overall, *N*=24 participants). Self-reports were reduced by principal components analysis to a single factor score of AMP- and MA-induced Positive Agency (ΔPA) in each participant. We found drug-induced ΔGlu related positively with ΔPA (ΔGluMA *r*=+.44, *p*<.05, *N*=21), with large effects in females (ΔGluMA *r*=+.52, *p*<.05; ΔGluAMP *r*=+.61, *p*<.05, *N*=11). States related to ΔGlu in females included rise in subjective stimulation, vigor, friendliness, elation, positive mood, positive affect (*r*’s=+.51 to +.74, *p*<.05), and alleviation of anxiety (*r*=−.61, *p*<.05, *N*=11). Self-reports correlated with DGlu to the extent they loaded on ΔPA (*r*=.95 AMP, *p*=5×10^−10^; *r*=.63 MA, *p*=.0015, *N*=11), indicating coherence of ΔGlu effects. Timing data indicated Glu shaped emotion both concurrently and prospectively, with no relationship to pre-MRS emotion (ΔGluAMP *r*=+.59 to +.65, *p*’s<.05; ΔGluMA *r*=+.53, *p*<.05, *N*=11). Together these findings indicate substantive, mechanistic contributions of neocortical Glu to positive agentic states in healthy individuals, most readily observed in women.

## INTRODUCTION

Glutamate (Glu) is an ancient compound that likely shapes conscious experience, well-being, and agency in everyday life. It is well established that Glu mediates excitatory neurotransmission, learning, memory, motor activity, is under homeostatic control and is excitotoxic when dysregulated (Magi et al, 2019). This scope would seem to argue against additional major, undiscovered roles for Glu in the brain. The evolutionary history of Glu, however, reveals rich diversity. Phylogenetically, Glu occurs at high concentrations in species that are extraordinarily distant - such as bacteria and humans - evidencing biologic roles predating divergence of prokaryotes and eukaryotes 2.7 billion years ago (Commichau et al, 2008; Cooper, 2000). Glu-like receptors occur in plants and animals, indicating common ancestry ~1.6 billion years ago (Chiu et al, 1999; Meyerowitz, 1999; Wang et al, 1999). Glu is thus ancient and ubiquitous, providing ample time and material for the evolution of multiple, overlapping functions in living systems. This history suggests additional undiscovered roles for Glu in healthy organisms, where rapid homeostasis readily obscures functional processes *in vivo*. Indeed, science over the last nine decades has repeatedly underestimated the functional diversity of Glu (Danbolt, 2021; Watkins and Jane, 2006). Glu thus very likely has further, unanticipated roles in health and well-being.

Emerging work in clinical neuroscience indicates neocortical Glu shapes the etiology and treatment of disorders of mood, motivation, and behavior. Glx, a combination of Glu and glutamate (Gln), is reduced in frontal brain in major depressive disorder (MDD) and is elevated in bipolar disorder (BPD) (Moriguchi et al, 2019; Scotti-Muzzi et al, 2021). Effective treatments for MDD such as electroconvulsive therapy, repetitive transcranial magnetic stimulation (rTMS), ketamine, and citalopram are associated with increase in Glx, Glu and Gln in frontal and temporal cortex, tracking MDD improvement (Gonsalves et al, 2022; Lener et al, 2017; Michael et al, 2003a, b; Milak et al, 2016; Pfleiderer et al, 2003). Moreover, rTMS with adjunctive D-cycloserine (100mg oral), a partial NMDA receptor agonist, yields greater alleviation of depressive symptoms than rTMS with placebo, indicating a role of Glu in recovery of positive affect (Cole et al, 2022). Euthymic states also correspond to heightened Glx, Glu and Gln in the ACC, and mood stabilizers alter Glu and Gln (Scotti-Muzzi et al, 2021; Soeiro-de-Souza et al, 2018). Further, individuals recovering from stimulant dependence report depressive symptoms that coincide with reduction in Glx in inferior frontal cortex (Bakhshinezhad et al, 2022; O’Neill et al, 2014). These data suggest contributions of neocortical glutamate to agentic states and amelioration of aversive states in clinical disorder.

Further insight is provided by drug challenge studies in healthy individuals, where phasic perturbation of neocortical Glu alters conscious experience. Here, single doses (30–40 mg oral) of memantine, an NMDA receptor antagonist, increase volunteers’ reports of feeling high, stimulated, forgetful, contented, lightheaded, detached, unreal, slow-motion, “buzzed”, and dizzy (Bisaga and Evans, 2004; Jackson et al, 2009). Single doses of D-cycloserine (50 mg oral) increase volunteers’ ratings of stimulation (Nesic et al, 2011). Low-doses of ketamine, a noncompetitive NMDA receptor antagonist, increase Gln in the ACC and reports of time distortion, dissociation, emotional blunting, cognitive disruption, excitement, and somatic activation (Coull et al, 2011; Krystal et al, 2005; Rowland et al, 2005). Conversely, the anesthetic propofol reduces Glu in motor cortex, sensory cortex, and thalamus alongside its effects on sedation (Zhang et al, 2009). These data suggest mechanistic contribution of neocortical Glu to visceral sensations, subjective states, and feelings of connection, attachment, and engagement.

**Figures 1:**
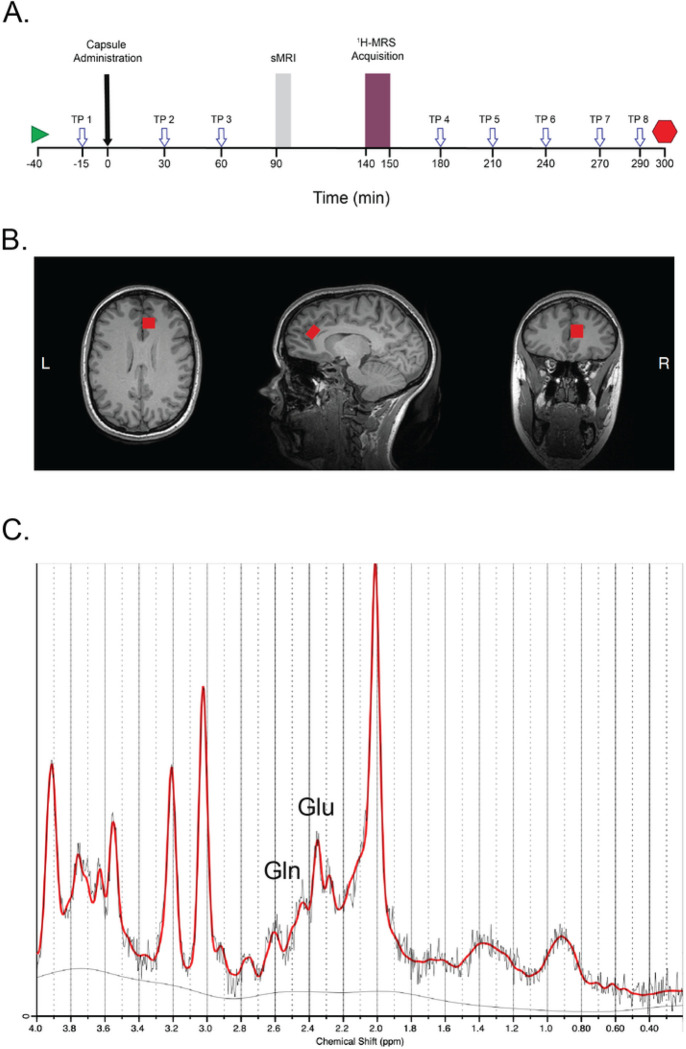
Experimental Approach **(A)** Experimental design and timing of test sessions. Sessions were 5.5 hours in duration (340 min total). X-axis denotes time relative to administration of the blinded study capsule at time 0 (black arrow). Participants entered the scanner 90 minutes after administration of the study capsule and structural MRI scan was conducted (denoted in gray shading). MR spectroscopy was conducted 140 to 150 minutes post-capsule (denoted in burgundy shading). Mood data were collected at half hour intervals (8 timepoints, TP) outside the scanner to assess subjective drug effects (open arrows, see methods for details). Participant arrival and departure times are indicated (start, stop symbols). **(B)** Voxel placement in dorsal anterior cingulate cortex (dACC). Left: axial; middle: sagittal; right: coronal views, respectively. Images are in neurological orientation (R=R). **(C)** Example MRS spectra with labeled peaks. The solid red curve overlay is the fitted spectrum from LCModel, and the raw data shown in light gray. Labeled peaks: Glu = glutamate, Gln = glutamine.

Motivated by the above data and history, we here investigate contributions of neocortical glutamate to conscious experience using drug challenge in healthy volunteers. This method provides experimental manipulation of neocortical Glu in individuals who are medically and psychiatrically well (White and Gonsalves, 2020; White et al, 2018). Toward this end, *d*-amphetamine (AMP), methamphetamine (MA) and placebo (PBO) were administered on three separate test days to healthy participants using a within-subjects, counterbalanced, double-blinded design, with each participant serving as their own control. Using this approach, we find AMP and MA produce phasic rise in dACC Glu, an effect most apparent in females (White et al, 2018). Informed by this finding and our prior work on drug effects and positive emotion (Grodin and White, 2015; Morrone et al, 2000; Weyandt et al, 2018; White, 2011, 2017; White *et al*, 2020; White and Gonsalves, 2021a; White et al, 2023; White et al, 2007; White et al, 2006), we here evaluate the relationship of dACC Glu and participants’ conscious experience, assessed by a large battery of validated self-report (SR) measures of subjective states at half hour intervals over the 5.5-hour period on each session (Fig. 1). This approach provides detailed information on conscious states, mood, emotion, metacognition, and visceral/somatic sensations in each participant, suitable for evaluation with phasic change in dACC Glu.

Our hypotheses were two-fold. First, we expected drug-induced change in neocortical Glu to predict the magnitude of drug-induced change in conscious states with motivational component, i.e., perceived and reported states of subjective stimulation, excitement, enthusiasm, and vigor. Second, we expected these effects to be more readily observed in female participants, due to their heightened glutamatergic response and increased vulnerability to stimulant dependence compared to males (White et al, 2018). The study thus provides new information on experimentally-induced change in neocortical Glu and its impact on subjective experience in well individuals.

## METHODS AND MATERIALS

### Procedures

This study was approved by the Institutional Review Board for research with human subjects at Brown University and the Memorial Hospital of Rhode Island and all participants provided informed consent. Procedures for recruitment, drug administration, MR structural imaging, MRS acquisition and metabolite quantification are published (details in Supplemental Methods, (White et al, 2021b; White et al, 2018). Drug effects on neurometabolites (White et al, 2018) and relationships among trait emotion and neurometabolites on PBO are published (White et al, 2021b). No adverse events were found for either of the drugs or PBO.

### Participants

Twenty-four participants completed MRS (*N* = 24, 12 female; (White et al, 2018)). Participants were 18–28 years of age (mean = 22.50 years, *SD* = 3.18, *N* = 24) and of normal body weight (mean BMI = 23.20, *SD* = 2.94; mean body weight = 144.91 pounds, *SD* = 18.12; mean height = 66.45 inches, *SD* = 4.06). Racial composition was 79% White, 17% Asian, and 4% African American. Ethnicity was 78% non-Hispanic and 22% Latino/Hispanic. Participants were well educated, with 8% reporting a high school diploma, 54% reporting some college education and 38% reporting a bachelor’s degree or beyond.

### Study Design, Drugs and Dosing

*d*-amphetamine sulfate (AMP, 20 mg oral), methamphetamine hydrochloride (MA; Desoxyn^®^, 20 mg oral), and placebo (PBO, dextrose) were administered in a counterbalanced, double blind, within-subjects crossover design. MRS imaging was conducted 140–150 m post-drug and PBO (Fig. 1, Supplemental Methods, White et al, 2018).

### Subjective Measures

Subjective states were assessed by six self-report instruments: the Positive and Negative Affect Schedule (Watson et al, 1988), Positive Activation Rating Scale (Morrone et al, 2000), Negative Activation Rating Scale (Weyandt et al, 2018), Visual Analogue Scales (Wewers and Lowe, 1990), Addiction Research Center Inventory (Haertzen, 1966), and Profile of Mood States (McNair and Droppleman, 1971). These self-report instruments provide information on subjective mood, valenced emotional states, psychoactive drug effects, visceral/somatic sensations, and metacognition at half-hour intervals across a 5.5-hour period on each session (Fig. 1; full details in Supplemental Methods) and have not been evaluated previously with dACC Glu (White et al, 2021b; White et al, 2018).

### MRS Quality Control and Analysis

Quality control entailed four steps. (a) MRS spectra from each session in each participant was fit using LCModel (Provencher, 1993), and visually inspected for quality (Fig. 1). (b) MRS voxels (placed by visual location on the right dACC, Fig. 1) were reconstructed in each anatomical scan and segmented using SPM12 and Gannet, providing information on voxel localization and segmentation (Ashburner and Friston, 2005; Harris et al, 2015). (c) MRS data were corrected for partial volume effects per formula [*1/(1- fCSF)] (Brandt et al, 2016; Larsen et al, 2016; Maltezos et al, 2014). (d) Data for individual metabolites were excluded where CRLB exceeded 20% *SD*. (e) Self-reports were evaluated for missing data (none missing). The procedures indicated self-report and MRS data were of high quality, yielding *N* = 24 for analysis of subjective states and *N* = 18–21 for analyses of neurometabolites.

### Statistical Analyses

#### Reduction of Self-report Data

**Figure 2:**
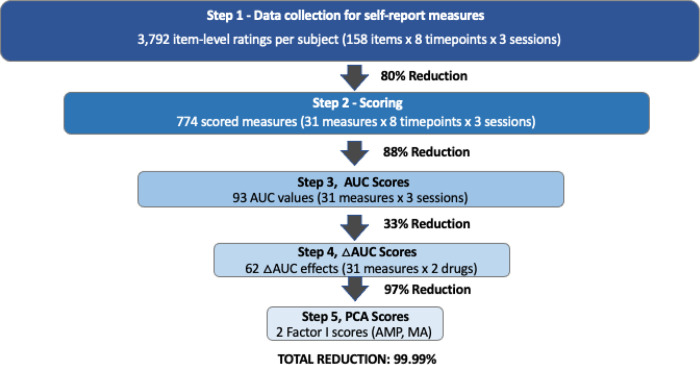
Data Reduction Data reduction procedure for within-subject self-report measures. AUC = area under the curve. ΔAUC = AUC on drug session minus AUC on placebo session. PCA = principal components analysis. AMP = *d*-amphetamine. MA= methamphetamine (Desoxyn^®^).

Subjective states were evaluated by 158 self-report items assessed on eight time points per session in each participant (AMP, MA, PBO; design in Fig. 1), yielding 3,792 item-level responses per participant (91,008 responses overall, *N* = 24). These data were reduced in four steps (Fig. 2; full details in Supplemental Methods). (1) Raw data were scored using standard methods of each instrument, yielding scored measures at each time point. (2) Area under the curve (AUC) values were calculated using the trapezoidal method across the eight time points of assessment on each session (AUCAMP, AUCMA, AUCPBO) (Leventhal et al, 2017). (3) Within-subject drug effects were calculated as ΔAUCAMP=AUCAMP minus AUCPBO, and ΔAUCMA=AUCMA minus AUCPBO. (4) Last, principal components analysis (PCA) of ΔAUCAMP and ΔAUCMA values were conducted, yielding a primary factor of subjective response to AMP and to MA (eigenvalue > 6.0, Figure S1). This factor (Factor I) summarizes participants’ subjective response to each drug, interpreted based on loadings greater than |.35|.

#### Statistical Tests of Hypotheses

##### Glu effects on Positive Agency.

Relationship of change in Glu and subjective states were evaluated using a correlation approach, with delta (Δ) scores of drug-induced changes in Glu entered as the predictor and Factor I score (z-score) of drug-induced change in Positive Agency entered as the dependent measure.

##### Contributions of biological sex.

Sex differences in the direction and magnitude of relationships were assessed by Fisher r-to-z transformation.

*Follow-up Analyses* were conducted to determine specificity, phenomenology, coherence, and timing of significant effects, as described below.

##### Specificity.

Relationships with change in Glx, Gln and Factor I response was evaluated jointly and separately by sex, to provide information on specificity of relationships with neurometabolites.

##### Phenomenology.

Relationship of ΔGlu and ΔAUC values (18 measures loading >|.35| on AMP and MA Factor I, Table 1) were evaluated using a correlation approach, providing information on specific subjective states related to Glu. This analysis was restricted to females, who accounted for significant effects at the group level.

##### Coherence.

Strength of ΔAUC measures’ relationships with ΔGlu (correlation coefficients with Glu) and with ΔPA (loadings on Factor I) were evaluated using a correlation approach. Analysis included measures loading >|.35| on Factor I to AMP and MA (Table 1) and was restricted to females (per significance criteria). This analysis provides information on coherence of ΔGlu effects on self-reports.

##### Timing.

Measures with significant drug x time interaction effects (POMS Vigor, POMS Friendliness, Table S3) were evaluated for time-dependent effects of ΔGlu. This analysis was restricted to females (per significance). ΔAUC values were calculated for timepoints (TP) prior to MRS (TP1 to 2), contiguous with MRS (TP3 to 4), and following MRS (TP4 to 8). Each binned response was evaluated with ΔGlu using a correlation approach, to inform timing of ΔGlu effects on emotion.

#### Manipulation and Validity Checks

Four sets of manipulation and validity checks were conducted to verify the efficacy of the study drugs, validity of participants’ self-reports, and validity of the follow-up timing bins calculations and analysis.

(1) Drug effects on scored self-reports were assessed by within-subjects, repeated-measures ANOVAs with two levels of drug (drug, PBO) and eight levels of time (TP1–8, Fig. 1). The analysis provides data on magnitude, direction, and timing of drug effects on self-report measures, with attention to loadings > |.35| (Table 1). (2) Drug Effects onΔAUC values (Table 1) were assessed by t-tests (1-tailed). Positive t-values indicate greater ΔAUC under drug than PBO; negative t-values indicate lesser ΔAUC under drug than PBO; t-values of zero indicate no difference. We expected increased ΔAUC (i.e., drug-induced rise; positive t-values vs. PBO) for measures of positive activated emotion, somatic sensations, and arousal; and reduced ΔAUC (i.e., drug-induced reduction; negative t-values vs. PBO) for measures of inattention, negative affect, and sluggishness. (3) Sex differences in self-reports and summary scores (ΔAUC values, PCA Factor I) were evaluated by independent samples t-tests (2-tailed). (4) Timing bins’ΔAUC scores were compared by paired-samples t-tests, with ΔAUC responses expected to rise over time (1-tailed).

### Power and Effect Size Estimation

Power analyses were conducted in G*Power 3.1.9 using an alpha of .05 (Cohen, 1988; Faul et al, 2009). Effect sizes (Cohen’s *d*) were calculated using the formula ((mean-0)/SD), with Cohen’s *d* values of .2 interpreted as small effects, .5 as medium effects, and .8 as large effects. Pearson correlations of .1 were interpreted as small effects, .3 as medium effects, and .5 as large effects (Cohen, 1988, 1992).

## RESULTS

### PCA Results

#### Factor I: Positive Agency.

Principal components analysis (PCA) of ΔAUCAMP and ΔAUCMA produced a primary factor of response to AMP and MA (Factor I; eigenvalue > 6.0, Figure S1), and interpreted based on loadings > |.35| (Table 1, below).

##### AMP.

Factor I to AMP had high positive loadings from POMS Positive Mood; PANAS Positive Affect; POMS Arousal, Elation, Vigor, Friendliness; ARCI Benzedrine Group; VAS Interested, Content, Stimulated; ARCI MBG, Amphetamine; VAS Elated; and PARS PA. There were high negative loadings from ARCI PCAG; POMS Confusion, Fatigue; ARCI LSD; POMS Depression, Anxiety, and Anger. The factor thus represents AMP-induced *Positive Agency* (ΔPAAMP), with an increase in positively valenced states incentive motivation (elation, vigor, positive mood, arousal, and interest) and a decrease in aversive and demotivated states (sedation, fatigue, depression, and anxiety) compared to PBO (Table 1).

##### MA.

Factor I to MA had high positive loadings from POMS Positive Mood; PANAS Positive Affect; POMS Elation; ARCI BG; POMS Friendliness, Vigor; ARCI MBG; POMS Arousal; ARCI A; VAS Interested, Stimulated, Elated, Want Alcohol, Content; and PARS PA, with moderate positive loading from ARCI M. There were high negative loadings from ARCI PCAG; POMS Fatigue, Confusion; and VAS Drowsy, with moderate negative loading from POMS Depression. The factor thus represents MA-induced *Positive Agency* (ΔPAMA), with an increase in positively valenced states of incentive motivation (positive emotion, arousal, vigor, interest, elation, positive mood) and a decrease in aversive and demotivated states (sedation, anxiety, depression, and fatigue) compared to PBO (Table 1).

##### Cross-drug effects.

Loadings were similar across drugs, with 18 measures loading greater than |.35| on ΔPAAMP and ΔPAMA (Table 1). Factor I scores for AMP and MA were positively correlated (ΔPAAMP, ΔPAMA*r* = + 0.61, 1-tailed *p* = 0.001, *N* = 24), indicating reproducibility of response and factor structure across the study drugs.

### Hypothesis Tests

#### Glu Effects on Positive Agency.

**Figure 3:**
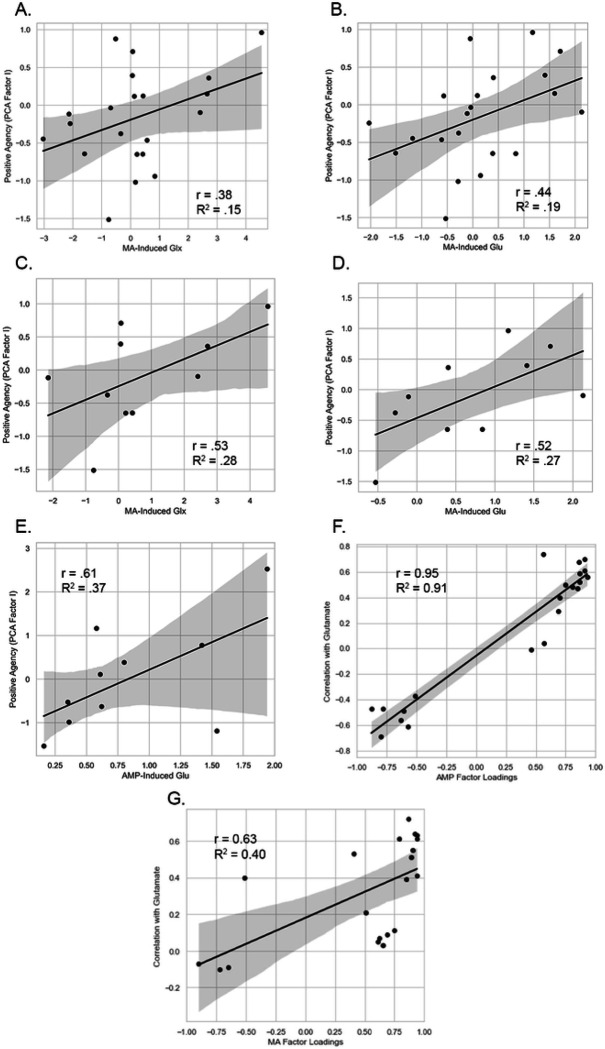
Δ Glu Effects on Positive Agency Relationship of Change in Glx and Glu with PCA Factor I and subcomponents. **(A)** MA-induced ΔGlx and Positive Agency, *N*=21 males and females, *p*<.05 **(B)** MA-induced ΔGlu and Positive Agency, *N*=21 males and females, *p*<.05. **(C)** MA-induced ΔGlx and Positive Agency, *N*=11 females, *p*<.05. **(D)** MA-induced ΔGlu and Positive Agency, *N*=11 females, *p*<.05. **(E)** AMP-induced ΔGlu and Positive Agency, *N*=11 females, *p*<.05. **(F)** Factor I loadings predict relationships with AMP-induced Glu, *r* = .95, *p*=5×10^−10^. The finding explains 91% of the variance in relationships of self-report measures and ΔGlu, *R*^2^ = .91. **(G)** Factor I loadings predict relationships with MA-induced Glu, *r* = .63, *p* = .0015. The finding explains 40% of the variance in the relationships of self-report measures and ΔGlu, *R*^2^ = .40. **Summary.** Panels A - E indicate positive relationship of drug-induced Glu, as assessed by change in Glx and Glu compared to PBO, and induced Positive Agency (Factor I scores). This effect was due to large effects in females (panels C - E). Panels F - G indicate the relationship of self-report measures and drug-induced change in glutamate. Individual self-report measures correlated with Glu to the extent they loaded on Factor 1 (Positive Agency).

MA. ΔGluMA and ΔGlxMA related positively to ΔPAMA in females and in the full sample (large, medium effects; Table 2, Fig. 3, Table S1). Findings in males were not significant (Table 2, Figure S2). AMP. ΔGluAMP related positively to ΔPAAMP in females, a large effect (Table 2, Fig. 3). ΔGluAMP and ΔGlxAMP findings in males and combined sample were not significant (Table 2, Table S1, Figure S2). These data indicate large effects of ΔGlu on ΔPA in females, accounting for effects at the sample level.

#### Contributions of Biological Sex.

Magnitude and direction of relationships of ΔGlu and ΔPA did not differ significantly between males and females (ΔGlxMA and ΔPAMA: *z*observed=.91, *p* = .18, n.s.; ΔGluMA and ΔPAMA: zobserved=.47, p = .32, n.s.; ΔGluAMP and ΔPAAMP: zobserved=1.37, p = .085, n.s.), indicating a lack of sexual dimorphism in ΔGlu effects on ΔPA.

### Follow-up Tests

#### Specificity.

ΔGlnMA and ΔGlnAMP were unrelated to ΔPAMA and ΔPAAMP, respectively (Table S1), indicating specificity of effects to Glu rather than Gln.

#### Phenomenology.

##### AMP.

ΔGluAMP related positively to rise in POMS Elation, Positive Mood, Friendliness, Arousal, and Vigor; VAS Elated; and PANAS Positive Affect (r’s from + .52 to + .74, p < .05), and negatively to POMS Confusion, Anxiety, and ARCI LSD; all large effects (*r*’s from − .56 to − .69, p≤.05 to .005; Table 2, Table S2). MA. ΔGluMA related positively to rise in POMS Elation, Positive Mood, Friendliness, and Vigor; ARCI A, M, BG, and MBG, all large effects (*r*’s from + .51 to + .72, *p*≤.05 to .01; ΔAUCMA in Table 2B, Table S2). Together these data indicate ΔGlu effects on elation, positive mood, friendliness, and vigor, with replication across AMP and MA.

#### Coherence.

Measures’ strength of relationship to Factor I (i.e., loadings on Factor I) predicted their strength of relationship to ΔGlu, a large effect (ΔGluAMP*r* = 0.95, *p* = 5×10^−10^ (1-tailed); ΔGluMA*r* = 0.63, *p* = .0015 (1-tailed)). Self-report measures thus related to ΔGlu to the extent they loaded on PA (Fig. 3F–G), evidence of coherence of ΔGlu effects across self-report instruments.

##### Timing.

###### AMP.

Δ GluAMP correlated positively to bin2 and bin3 Vigor and Friendliness (r’s from + .59 to + .65, *p* < .05). There was no relationship to bin1 (Table 2). These data indicate ΔGluAMP predicted current and subsequent emotion (Fig. 1; Figure S3). MA. ΔGluMA correlated positively to bin 3 Friendliness (*r* = + .53, *p* < .05). There was no relationship to bins1 or 2 (Table 2). These data indicate ΔGluMA predicted subsequent emotion (Fig. 1; Figure S3).

### Manipulation & Validity Checks

#### Drug efficacy.

Drug effects on self-reports and summary scores (i.e., scored measures, ΔAUC values, Factor I scores) were highly significant. These data indicate efficacy of the study drugs and validity of summary score calculations, quality control and data reduction procedures (Supplemental Results, Tables S3-S4).

#### Subjective responses & timing bins.

Δ AUC values differed by bin, with rise in value over time (Supplemental Results, Figure S3). ΔAUC and PCA Factor I responses did not differ by sex (Supplemental Results, Table S5). These data indicate feasibility of time-dependent prediction of emotion by ΔGlu, and an overall lack of sex differences in the subjective response to AMP and MA.

#### Statistical Power.

The sample of 24 had high power (1-β = .96) to detect large effects (*d* = .80), adequate power (1-β = .80) to detect medium effects (*d* ≥ .60), and low power (1-β = .16) to detect small effects (*d* = .20). There was high power (1-β = .83) to detect large correlations (*r* ≥ .5) and low power (1-β ≤ .42) to detect small to medium correlations (*r* ≤ .3).

## DISCUSSION

There were six sets of findings. Experimentally-induced change in neocortical Glu was positively related to rise in reports of positive agentic states in the sample. These effects were specific to Glu and unrelated to Gln, evidence of specificity. Effects were significant in females, who accounted for results at the sample level. Further, self-reports related to DGlu to the extent they loaded on agency (DPA, Factor I), indicating cohesion across measures. Follow-up analyses indicated DGlu specifically related to rise in subjective stimulation, vigor, friendliness, elation, positive mood, positive affect, and alleviation of anxiety. Timing analyses indicated Glu predicted current and later emotion. Together these results indicate acute rise in neocortical Glu relates to rise in positively valenced agentic emotion, with capacity for concurrent and prospective prediction. Potential mechanisms and implications are below.

Our main finding was a robust positive relationship of experimentally-induced DGlu and positive agentic emotion in females (Table 2, Figure 3). This effect was large in size and occurred for AMP and MA, indicating reproducibility across study drugs and test days (Figure 3). Induced emotion was independent of Gln (Table S1), indicating specificity of glutamate. Effects in females accounted for patterns at the group level. Thus our major finding was the strong, specific, reproducible, positive relationship of rise in neocortical Glu and positive agentic states in females.

This finding is consistent with prior work indicating pharmacologic- and recovery-related reduction in Glu relates to reduction in agentic phenomena. For instance, in rodents pharmacologic blockade of glutamatergic receptors - via microinjection of mGlu2/3 antagonist LY341495 to the nucleus accumbens - reduces behavioral markers of appetitive motivation and reward ‘liking’ (Richard and Berridge, 2011). In adults recovering from stimulant dependence, early drug abstinence is characterized by increased depression and reduced Glx and Glu in posterior cingulate, precuneus, and right inferior frontal cortex (O’Neill *et al*, 2014). Our findings complement and extend this work, indicating acute increase in neocortical Glu precedes and contributes to positive agentic states in healthy individuals.

While Glu-emotion effects were significant in females, relationships did not differ in magnitude or direction as a function of biological sex. This indicates a lack of sexual dimorphism in subjective effects of Glu. Females’ larger glutamatergic response to AMP and MA compared to males (White *et al*, 2018) provides a larger predictable range of experimentally-induced Glu in females. Similarly, truncated or mixed Glu response in males (White *et al*, 2018) reduces predictable range and statistical significance of relationships in males. Given the small sample size of males and females in the present design, investigation of gender differences in larger samples is warranted.

Follow-up tests indicated Glu affected specific emotional states. Rise in Glu related to increase in subjective stimulation, vigor, friendliness, elation, positive mood, positive affect (*r*’s=+.51 to +.74, all *p*<.05) and alleviation of anxiety (*r*=−.61, *p*<.05). Vigor, friendliness, elation, and positive mood were associated with AMP- and MA-induced DGlu (Table 2, Table S2), evidence of replicability. In addition DGlu predicted self-reports to the extent these measures loaded on the factor of agency (Factor I; *r*=.95, *p*=5×10^−10^ for AMP; *r*=.63, *p*=.0015 for MA; Figure 3). Thus DGlu related to self-reports to the extent they involved an incentive motivational component (i.e., a positive agentic response). These data demonstrate coherence of Glu effects on subjective states, with findings generalizable across study conditions (AMP, MA), measures (vigor, friendliness, elation, positive mood), and data reduction approaches (Factor 1, AUC; Figure 2).

Effect timing was informative, with drug-induced change in Glu shaping both concurrent and later reports of positive emotion (DGlu *r*=+.59 to +.65, *p*’*s*<.05 with AMP; DGlu *r*=+.53, *p*<.05 with MA). Change in neocortical Glu preceded or co-occurred with self-reports (timing in Figure 1), indicating contribution of Glu to current and subsequent emotion. Positive emotions stayed higher throughout the period of testing, lasting five hours post-drug and 2.5 hours post-Glu assessment (Figure 1, Table 2, Figure S3). This duration of effects has therapeutic implications, as Glu may provide a marker to target and personalize interventions in MDD, substance use disorder, and to improve overall well-being during periods of health. The findings are also consistent with prior work indicating drug-induced Glu predicts extent and magnitude of drug high and drug liking, and the positive relationship of dACC Glu and trait measures of positive agency at rest (White *et al*, 2021b; White *et al*, 2018).

Together these findings are consistent with clinical and preclinical literature that demonstrate heightened vulnerability to psychostimulants in females. In animal studies, females show enhanced behavioral sensitization to psychostimulants compared to males (McCormick *et al*, 2005; Van Swearingen et al, 2013). In human studies, drug users who are female develop psychostimulant dependence more rapidly and to greater extent than males (Anker and Carroll, 2011). National epidemiologic data further indicate females’ earlier chronological age of first use of cocaine and amphetamine, and females’ more rapid progression from initial use to drug dependence compared to males (Becker and Hu, 2008). Responses to psychostimulants are thus modulated by biological sex in ways that facilitate females’ rapid acquisition and persistence of drug dependence. Our findings indicate a role of neocortical glutamate in subjective experience after drug ingestion, with pronounced effects in females. These subjective effects may shape both the etiology and trajectory of stimulant dependence and MDD. Heightened glutamate-mediated learning of contextual cues, drug-cue associations, and glutamate-mediated reward processing in females would contribute to more rapid acquisition and severity of drug dependence in females compared to males. In the context of MDD, our findings advance Glu as a novel treatment target for medication and adjunctive treatment for positive emotion recovery (Cole *et al*, 2022).

The present study has both strengths and weaknesses. Strengths include use of a within-subjects, placebo-controlled crossover drug challenge design; assessment of subjective states through multiple self-report instruments at eight time points on three test sessions per participant; and rigorous procedures for data quality and data reduction. Use of within-subjects, repeated-measures assessment of states provide deep phenotyping of subjective states, emotion, visceral and somatic sensations, and metacognition suitable for analysis with experimentally-induced change in Glu.

Limitations included the modest sample size, low statistical power to detect sexual dimorphism in Glu effects on emotion, and relatively high CRLB uncertainty for Gln due to the PRESS acquisition. While relative CRLBs are common practice in reporting MRS data quality and 20% is a common threshold, this threshold is likely overly conservative for Glu and Gln (Kreis, 2016). As Gln is difficult to distinguish from Glu at the present TE at 3T, future studies should implement acquisition parameters that more effectively differentiate Gln from Glu. The present measures of Glx, Glu and Gln include both metabolic pools and neurotransmitter levels of Glu and Gln, as MRS supplies data on the total tissue metabolite within the voxel. Future work can utilize larger samples, assess ovarian, testicular, and adrenal hormones; epigenetics; sex-dependent gene expression; and social constructions of gender.

In summary, we here identify a robust positive relationship of acute rise in dACC glutamate and positive agentic subjective states in healthy females. Timing was concurrent and prospective, with no relationship to pre-MRS emotion. To our knowledge, this is the first demonstration that acute change in glutamatergic compounds in human cortex alters a broad range of positive agentic states in well individuals. The study thus indicates a substantive, mechanistic contribution of neocortical Glu to positive agentic emotion that is readily observed in females.

## Figures and Tables

**Table 1:** Principal Components Factor I

ΔAUC Self-Report Measures	*Positive Agency* *AMP*	*Positive Agency* *MA*
**PANAS: Positive Affect**	**.91**	**.94**
PANAS: Negative Affect	.08	−.09
**PARS: Positive Activation**	**.46**	**.51**
NARS: Negative Activation	−.01	.02
**ARCI: PCAG**	**−.88**	**−.90**
**ARCI: Benezedrine**	**.85**	**.92**
**ARCI: Amphetamine**	**.57**	**.79**
**ARCI: MBG**	**.69**	**.87**
ARCI: LSD	**−.63**	−.12
ARCI: Marijuana	−.14	**.41**
POMS: Anxiety	**−.57**	−.27
**POMS: Depression**	**−.61**	**−.43**
POMS: Anger	**−.51**	−.04
**POMS: Vigor**	**.87**	**.89**
**POMS: Fatigue**	**−.78**	**−.72**
**POMS: Confusion**	**−.80**	**−.65**
**POMS: Friendliness**	**.86**	**.90**
**POMS: Elation**	**.87**	**.94**
**POMS: Arousal**	**.91**	**.85**
**POMS: Positive Mood**	**.93**	**.94**
**VAS: Stimulated**	**.70**	**.69**
**VAS: Interested**	**.81**	**.75**
VAS: Queasy	−.12	−.11
**VAS: Content**	**.75**	**.61**
VAS: Drowsy	−.33	**−.51**
VAS: Anxious	.03	−.33
**VAS: Elated**	**.56**	**.65**
VAS: Nauseated	−.25	−.16
VAS: Sedated	−.29	−.02
VAS: Hungry	.19	.04
VAS: Want Alcohol	.17	**.62**

*Legend:* Factor I loadings greater than |0.35| are in **bold.** Measures loading greater than |0.35| on both factors are in gray. AUC calculated as the area under the curve (AUC) on the drug session minus AUC on the PBO session (details in methods).

Positive Agency = PCA Factor I response to AMP and MA, respectively.

AMP = *d*-amphetamine, MA = methamphetamine (Desoxyn^®^).

*N* = 24 healthy volunteers.

**Table 2:** ΔGlu Effects on Positive Agency

A.	Positive Agency (AMP)	Positive Agency (MA)
ΔGlu (f)	**.61** *	**.52** *
ΔGlu (m)	−.03	.31
ΔGlu (m + f)	.27	**.44** *

B.	Vigor (ΔAUC_AMP, MA_)	Friendliness (ΔAUC_AMP, MA_)	Elation (ΔAUC_AMP, MA_)	Positive Mood (ΔAUC_AMP, MA_)
ΔGlu_AMP_ (f)	**.59** *	**.68** **	**.52** *	**.56** *
ΔGlu_MA_ (f)	**.51** *	**.55** *	**.63** *	**.61** *

C.	Vigor 1 (Δ AUC_Bin1_)	Vigor 2 (Δ AUC_Bin2_)	Vigor 3 (Δ AUC_Bin3_)	Friendliness 1 (Δ AUC_Bin1_)	Friendliness 2 (Δ AUC_Bin2_)	Friendliness 3 (Δ AUC_Bin3_)
ΔGlu_AMP_ (f)	.28	**.59** *	**.59** * ^b,c^	.48	**.65** *	**.61** * ^∞^
ΔGlu_MA_ (f)	.51	.46	**.33** ^a^	.29	.44	**.53** *
*Legend.*						
2A. Relationship of change in Glu and Positive Agency (Factor I), *N* = 11 females, *N* = 9,10 males for AMP, MA, respectively; combined sample *N* = 20,21 for AMP, MA, respectively.						
2B. Consistency in ΔGlu Effects on self-report measures (details in SI). *N* = 11 females.						
2C. Rise in ΔGlu Effects on self-reports over time. *N* = 11 females.						

Abbreviations: AMP = *d*-amphetamine. MA= methamphetamine (Desoxyn^®^).

f = females. m = males. m+f = males and females.

ΔAUC = difference in area under the curve (AUC) for self-reports on the drug session minus the AUC for self-reports on PBO session.

ΔGlu_AMP_ = AMP-induced change in glutamate vs. PBO.

ΔGlu_MA_ = MA-induced change in glutamate vs. PBO.

Bin 1= timepoints prior to MRS (timepoints 1–3, Figure 1).

Bin 2= timepoints contemporaneous with MRS (timepoints 3–4, Figure 1).

Bin 3= timepoints following MRS (timepoints 4–8, Figure 1).

Significant findings are in **bold.**

** p ≤.01

* p ≤05.

a *p* < .005 Bin 1 vs. Bin 3

b *p* < .05 Bin 1 vs. Bin 3

c *p* < .05 Bin 2 vs. Bin 3

¥ *p* < .10 Bin 1 vs. Bin 3.

*Contributions of Biological Sex.* Magnitude and direction of relationships of ΔGlu and ΔPA did not differ significantly between males and females (ΔGlx_MA_ and ΔPA_MA_: *z*_observed_=.91, *p* = .18, *n.s.*; ΔGlu_MA_ and ΔPA_MA_: *z*_observed_=.47, *p* = .32, *n.s.*; ΔGlu_AMP_ and ΔPA_AMP_ : *z*_observed_=1.37, *p* = .085, *n.s.*), indicating a lack of sexual dimorphism in ΔGlu effects on ΔPA.
